# Impact of COVID-19 pandemic on care of maintenance hemodialysis patients: a multicenter study

**DOI:** 10.1007/s10157-024-02495-6

**Published:** 2024-05-03

**Authors:** Yasmine Salah Naga, Ahmed El Keraie, Samar Samy Abd ElHafeez, Rowan Saad Zyada

**Affiliations:** 1 Nephrology Unit, Internal Medicine department, Faculty of Medicine, Alexandria University, Alexandria, Egypt; 2 Epidemiology Department, High Institute of Public Health, Alexandria University, Alexandria, Egypt; 3 Kidney and Urology Centre, 347 Gamal Abd El Naser Street, Montaza, Alexandria, Egypt

**Keywords:** COVID-19, Pandemic, Lockdown, Dialysis

## Abstract

**Background:**

The COVID-19 pandemic posed a challenge to hemodialysis (HD) patients. While most outpatient and elective medical services stopped during lockdown, HD patients continued to visit their dialysis centers. We aimed to assess how the initial phase of the pandemic affected patient care by comparing dialysis adequacy and other parameters of patient care before and during the first 10 months of the COVID-19 pandemic.

**Methods:**

In a retrospective multi-center observational study, all adult dialysis patients in five dialysis centers in Alexandria, Egypt were included. Dialysis adequacy, missed sessions, laboratory parameters and hospitalization were recorded. Data of the 10 months before and the 10 months after the pandemic were compared and predictors of adequacy were determined.

**Results:**

In the 388 HD patients included in the study, the number of missed sessions was higher during the pandemic with peaks during the first and second wave of the pandemic. The ratio of patients to nurses, phosphorus and parathormone levels were significantly higher during the pandemic, while urea reduction ratio, *Kt/V*, hemoglobin, calcium and albumin levels were significantly lower. In patients who reported difficult accessibility, missed HD sessions were higher during lockdown. Hospital admissions doubled during the pandemic, with COVID-19 infection being the main cause (45.5%). Number of patients per nurse and interdialytic weight gain were predictors of inadequate dialysis.

**Conclusion:**

The COVID-19 pandemic and its related lockdown negatively affected multiple aspects of dialysis patient care. Continued access of optimum care in dialysis patients should be a priority in any future mass events.

## Introduction

In the beginning of 2020, Severe Acute Respiratory Syndrome Coronavirus 2 (SARS-CoV-2) was identified as the causative agent of pneumonia outbreak in China. Within a short period of time, thousands of humans all over the world were infected as a result of high viral transmissibility and abundance of international travel. By March 11, 2020, the world health organization (WHO) declared coronavirus disease of 2019 (COVID-19) a global pandemic [1]. To limit its spread, many countries applied a lockdown which posed unprecedented health, social, economic, and environmental challenges [2], In Egypt, the first case of COVID-19 was reported on 14th February. With increasing number of cases, a suspension of public gatherings was implemented on March 19, 2020 with suspension of all flights, closure of schools, universities, mosques and churches to limit the outbreak of the coronavirus [3]. A night curfew was also imposed with these restrictions lasting till 27th June 2020 [4]. Campaigns of “Stay home, stay safe” and adopting social distancing were launched to increase public awareness of COVID-19 symptoms and preventive measures [3].

The impact of the COVID-19 pandemic and subsequent restrictions on healthcare exceeded the direct viral morbidity and mortality to affect the routine inpatient and outpatient care for chronic patients and elective procedures. The continued interruption of supply chain of medications and other medical equipment resulted in resource limitation and critical shortfalls. Priority in resources and health professional allocation was given to critical care units and isolation hospitals treating COVID-19 patients [5].

For hemodialysis (HD) patients, the COVID-19 pandemic represented a special challenge. During lockdown, there was difficult accessibility to HD units during roaming ban hours, and use of public transport services added infection exposure risk [6]. Many patients voluntarily missed their HD sessions for their fear of infection [7]. During dialysis, patients had to stay in close contact with other patients and dialysis staff increasing their risk of contracting infection. Some HD units decreased the duration of HD sessions to allow separation of patients and to apply screening and disinfection protocols. There were also shortages of physician and nursing workforce as they were debuted in isolation hospitals or were quarantined. Routine follow-up of dialysis adequacy, laboratory investigations, and transplant preparation were postponed [8]. In hospital-based HD units, dialysis-requiring COVID-19 patients with acute kidney injury were an added burden on HD units [9].

At the third near the fourth anniversary of the COVID-19 pandemic, some experts advocated the declaration of the end of the pandemic and its evolution to an endemic disease while others said it may be too early. At the media briefing—5 May 2023, the WHO general director declared “COVID-19 has left—and continues to leave—deep scars on our world’’ [10]. Although this pandemic came to an end, it has exposed a need for better preparation for possible future pandemics. Not only pandemics, but other local and global disasters such as earthquakes, floods, and wars may have a huge impact on patients with chronic illness who need continuous medical services [11].

The impact of COVID-19 on different aspects of care of end stage kidney disease (ESKD) patients in HD units remains largely unexplored. In this context, we conducted a multi-center retrospective observational study to compare hemodialysis adequacy and other parameters of patient care before and during the first 10 months of the COVID-19 pandemic in Alexandria, Egypt.

## Methods

### Study design and setting

This study is a retrospective multi-center cohort that included all adult ESKD patients (> 18 years old) maintained on HD for at least 3 months before the COVID-19 pandemic in five HD units in Alexandria, Egypt; El-Mowasah University Hospital, Alexandria University Student Hospital, Smouha University Hospital, Abu-Quir General Hospital and Kidney and Urology Center (3 university hospitals, one public sector hospital and one private sector hospital, respectively). Patients who started HD after the COVID-19 pandemic were excluded.

### Outcomes

Primary outcome: compare the hemodialysis adequacy parameters before and during the COVID-19 pandemic.

Secondary outcomes: address the impact of curfew on dialysis adequacy parameters.

### Data collection and study variables

Relevant variables were collected from the medical records of each unit as well as from personal interviews with patients including demographics, medical, drug and dialysis history during the first 10 months of the COVID-19 pandemic (from March 2020 till December 2020) and the 10 months before the pandemic (from June 2019 to February 2020).

The number of healthcare working staff in each unit, average duration of HD session per month, mean interdialytic weigh gain (IDWG), any report of difficult accessibility to HD unit during lockdown period, any vascular access complication, and number of hospital admissions, causes and duration were recorded. The number of missed HD sessions per month was reported if the patient missed any scheduled session regardless of being admitted or outpatient, and regardless of the place of dialysis. Patients on thrice weekly dialysis are expected to get 13 sessions per month and patients on twice weekly dialysis are expected to get 9 per month. If the patient did not get any session due to any reason, we reported a missed session.

Dialysis adequacy before and during the COVID-19 pandemic were assessed using laboratory values including monthly measured urea reduction ratio (URR) [12], monthly calculated *Kt/V* [12] (from the pre-dialysis to post-dialysis urea ratio (R), the weight loss (UF), session length in hours (*t*), and anthropometric or modeled volume (V) using the equation: *Kt/V* = In (*R* − 0.008 × *t*) + (4 − 3.5 × *R*) × 0.55 *UF/V*) [13]. Monthly hemoglobin levels [14], calcium and phosphorus measured at 3 months interval [15], albumin and parathyroid hormone level (PTH) [15] measured at 6 months interval were also recorded and the average during the 10 months before and after the pandemic were compared.

### Statistical analysis

Quantitative variables were summarized as mean ± standard deviation (SD) for normally distributed data or median [interquartile range (IQR)] for non-normally distributed data. The data distribution was checked using visual identification of a normal distribution by QQ plot. Qualitative variables were presented as percentages and frequencies. Paired *t*-test and Wilcoxon-rank test were used to compare quantitative variables, according to normality distribution. Chi-square test with Fisher exact correction when more than 20% of the cells have expected count less than 5 was used to compare for categorical variables. The McNemar test was used for paired nominal data. Multivariate logistic regression was performed to identify predictors of inadequate dialysis. Statistical analyses were performed using SPSS version 21 and *p*-value < 0.05 was considered significant.

## Results

Data of 388 adult ESKD patients pertaining to the period from June 2019 to December 2020 were collected from five HD units in Alexandria Governorate, three of which are university hospitals [El-Mowasah University Hospital (*n* = 143), Alexandria University Student Hospital (*n* = 64), and Smouha University Hospital (*n* = 11)], one ministry of health hospital (Abu-Quir general hospital, *n* = 100) and one private hospital (Kidney and Urology Center, *n* = 70).

The starting number of patients 10 months before the start of COVID-19 pandemic was 411 patients, out of which 23 died before the pandemic start (5.59%). During the first 10 months of the pandemic, 29 patients died (7.47%), 24 were COVID-19 related (82%) and 5 were non-COVID-19 related (17%). The relative risk (RR) of COVID-19 pandemic to increase mortality was 1.34.

### Participants characteristics

(Table 1) shows that the average age of the study participants was 51.6 ± 15.53 years, 56.4% were males. The most common cause of ESKD was hypertension (30.2%) followed by diabetic kidney disease (DKD) (24.7%) and chronic glomerulonephritis (CGN) (12.9%). The most common comorbidities were hypertension (80.2%), ischemic heart disease (IHD) (37.1%) and diabetes (25%). The median duration for HD was 4 years (IQR 2.0–9.50). Most patients were receiving thrice weekly HD sessions (84%). The vascular access for HD was mainly arteriovenous fistula (AVF) (86.3%) followed by tunneled-cuffed catheters (12.6%) and arteriovenous graft (AVG) (1.1%). Seven percent of the patients were receiving immunosuppressive medications, such as steroids, for associated autoimmune diseases, 10.6% were taking RAAS blockers including angiotensin converting enzyme inhibitors (ACEis) and angiotensin receptor blockers (ARBs) for blood pressure control.

**Table 1:** Baseline clinical characteristics of all patients

Clinical history	Total (*n* = 388)
Sex	
Male	219 (56.4%)
Female	169 (43.6%)
Age (years)	
Mean ± SD	51.61 ± 15.53
Cause of ESKD	
HTN	117 (30.2%)
DKD	96 (24.7%)
CGN	50 (12.9%)
Chronic pyelonephritis	30 (7.7%)
ADPKD	17 (4.4%)
Others	78 (20.2%)
Comorbidities	
HTN	311 (80.2%)
IHD	144 (37.1%)
DM	97 (25.0%)
HF	36 (9.3%)
COPD	31 (8.0%)
Autoimmune disease	24 (6.2%)
Hepatic disease	19 (4.9%)
Malignancy	7 (1.8%)
Vintage of HD in years	
Median	4.0
IQR	2.0–9.50
HD Frequency/weeks	
1/week	7 (1.8%)
2/week	55 (14.2%)
3/week	326 (84.0%)
Access	
AVF	335 (86.3%)
Cuffed-tunneled catheters	49 (12.6%)
AVG	4 (1.1%)
Active drug history	
Immuno-suppression	27 (7.0%)
RAAS blocker	41 (10.6%)

*IQR* Inter quartile range, *SD* Standard deviation, *AVF* arteriovenous fistula, *AVG* arteriovenous graft, *HTN* hypertension, *DKD* diabetic kidney disease, *CGN* chronic glomerulonephritis, *ADPKD* autosomal dominant polycystic kidney disease, *DM* diabetes mellites, *IHD* ischemic heart disease, *HF* heart failure, *COPD* chronic obstructive pulmonary disease, *RAAS* *blocker* renin–angiotensin–aldosterone system blocker

### Comparison between HD quality and adequacy before and during COVID-19 pandemic

The comparison of HD parameters before and during the pandemic are shown in (Table 2). During the 10 months before the COVID-19 pandemic, the total number of missed HD sessions for all patients was 1258 which represented 2.7% of their expected sessions. This number increased to 1433 HD sessions which represented 2.99% of their expected sessions during the first 10 months of pandemic.

**Table 2:** Comparison between HD parameters and adequacy before and during the COVID-19 pandemic

	Before COVID-19 pandemic	During COVID-19 pandemic	*p* value
Monthly missed HD sessions per patient			
Median (IQR)	2.0 (0.0 – 4.0)	2.0 (0.0 – 5.0)	0.019*
No. of HD patients/nursing staff			
Mean ± SD	4.10 ± 1.14	4.52 ± 1.48	< 0.001*
IDWG			
Median (IQR)	3.0 (2.50 – 3.50)	3.0 (2.50 – 3.70)	< 0.001*
URR%			
Mean ± SD	66.34 ± 5.84	63.48 ± 6.58	< 0.001*
URR ≥ 65%	196 (60.5%)	143 (44.7%)	< 0.001*
KT/V			
Mean ± SD	1.23 ± 0.21	1.15 ± 0.23	< 0.001*
Vascular access complications	11 (2.8%)	19 (4.9%)	^McN^*p* = 0.186

*IDWG* inter-dialytic weight gain, *HD* hemodialysis, *URR* urea reduction ratio

p: *p* value for comparing between before COVID-19 and During COVID-19**,** *McN* McNemar’s test

*Statistically significant at *p* ≤ 0.05

**Fig. 1:**
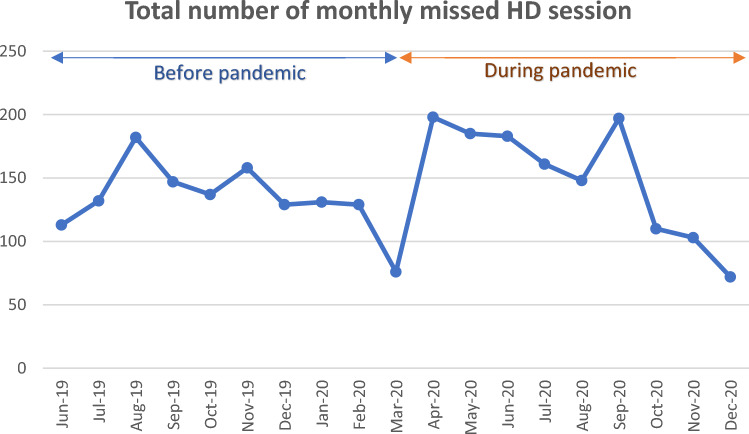
Distribution of monthly missed HD sessions over the study period in total sample

The number of missed sessions reached its maximum during the months of curfew; April, May, and June 2020 and a second peak was observed in September 2020 which coincides with the second wave of the COVID-19 pandemic in Egypt (Fig. 1). In all units, the standard duration of each HD session was 4 h, except in Abu-Quir unit, where the duration of HD sessions was decreased to 3 h during curfew months as most of patients depended on public transportation, so needed to finish their sessions before curfew hours. The mean number of HD patients per working nurse staff was significantly lower before the pandemic (4.10 ± 1.14) than during the pandemic (4.52 ± 1.48) (*p* < 0.001).

There was a significant difference between median IDWG before and during the COVID-19 pandemic (*p* < 0.001). Of the total 388 patients, 11 patients had at least one vascular access complication before the pandemic (2.8%) and 19 patients during the pandemic (4.9%).

### Laboratory parameters

**Fig. 2:**
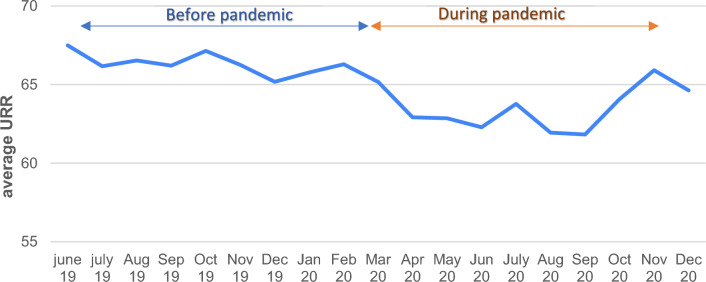
Monthly variation in URR during follow-up period

**Fig. 3:**
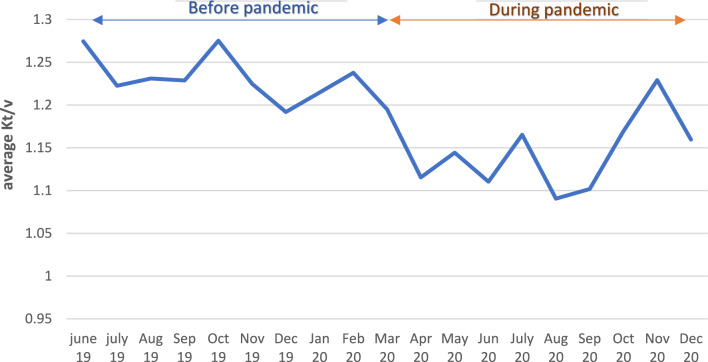
Monthly variation in Kt/V during follow up period

The mean URR during the pandemic (63.48% ± 6.58) was significantly lower than the mean URR before the pandemic (66.34% ± 5.84). (*p* < 0.001). 60.5% of total patients had URR% within target limit (more than 65%) before the pandemic while only 44.7% had URR% within target limit during the pandemic. The variation of monthly mean URR% over the study period is shown in (Fig. 2). The lowest readings were during the months of lockdown and continued till September 2020. The mean Kt/V during the pandemic (1.15 ± 0.23) was also significantly lower than the mean Kt/V before the pandemic (1.23 ± 0.21) (*p* < 0.001). The variation of monthly Kt/V over the study period is shown in (Fig. 3).

The median Hb level was significantly lower during the pandemic (9.96 g/dl (IQR 8.98–10.7) vs 10.20 g/dl (IQR 9.35–10.98), *p* < 0.001). Regarding markers of chronic kidney disease-associated mineral bone disease (CKD-MBD), calcium levels were significantly lower, phosphorus levels were significantly higher and PTH was significantly higher during the pandemic (*p* = 0.005, 0.033 and < 0.001, respectively). Serum albumin levels were also significantly lower during the pandemic (*p* < 0.001) (Table 3).

**Table 3:** Comparison between the average laboratory parameters before and during the COVID-19 pandemic

	Before COVID-19 pandemic	During COVID-19 pandemic	*p* value
Hb (g/dl)			
Median (IQR)	10.20 (9.35–10.98)	9.96 (8.98 – 10.78)	< 0.001*
Total calcium (mg/dl)			
Median (IQR)	8.77 (8.30–9.20)	8.67 (8.10 – 9.13)	0.005*
Phosphorus (mg/dl)			
Mean ± SD	5.46 ± 1.30	5.60 ± 1.37	0.033*
Albumin (g/dl)			
Mean ± SD	4.08 ± 0.37	3.93 ± 0.39	< 0.001*
Parathyroid hormone (pg/ml)			
Median (IQR)	320.50 (179.0–583.0)	352.0 (190.0–600.0)	< 0.001*
Urea (mg/dl)			
Mean ± SD	150.94 ± 7.58	151.22 ± 14.27	0.005*

### Impact of accessibility to HD units on dialysis adequacy

Only 23 patients (6%) reported difficult accessibility to the HD unit during curfew. All of them were assigned to the night HD shift that included time during curfew periods. To assess the impact of accessibility problems on quality of HD, we compared the laboratory parameters of dialysis adequacy and total number of missed HD sessions during the 3 months of curfew and the following 3 months in this group of patients (Table 4). There was a significant decrease in the total number of missed HD sessions after curfew lift (*p* = 0.001). URR% and mean hemoglobin levels also improved after curfew lift but not significantly.

**Table 4:** Comparison HD adequacy parameters during and after curfew in patients who reported difficult accessibility to HD units (*n* = 23)

	Difficult accessibility to HD (*n* = 23)		*p*
	During Curfew	After Curfew	
No. of monthly missed HD sessions per patient [Median (IQR)]	2.0 (0.0 – 5.50)	0.0 (0.0 – 1.50)	0.001*
URR (Mean ± SD)	58.12 ± 6.40	58.63 ± 5.67	0.711
Kt/V (Mean ± SD)	1.0 ± 0.26	0.99 ± 0.16	0.795
Hemoglobin (g/dL) [Median (IQR)]	9.50 (8.32 – 10.45)	9.93 (8.97 – 10.72)	0.626

p: *p* value for comparing between before COVID and after COVID

*Statistically significant at *p* ≤ 0.05

### Hospital admissions

**Fig. 4:**
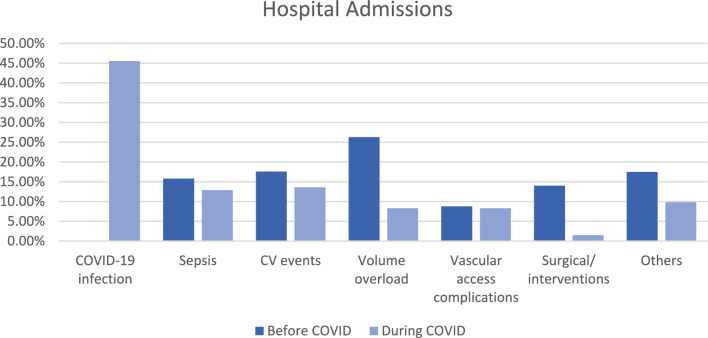
Comparison between causes of hospital admissions and ER visits before and during COVID-19 pandemic in total sample

The number of hospital admissions during the 10 months before COVID-19 pandemic was 57, while it was 132 admissions during the first 10 months of the pandemic. COVID-19 infection represented the most common cause for hospital admission during the pandemic period (45.5%) followed by cardiovascular (CV) events (13.6%) and sepsis (12.9%). Before the pandemic the most common cause for hospital admission was volume overload (17.6%) followed by CV events (17.6%) and sepsis (15.8%). The rate of surgical and elective interventions during the pandemic was significantly lower (*p* = 0.001). The presentation with signs of volume of overload was also lower than before the pandemic (*p* = 0.001). The median days of hospital stay during the pandemic was higher than before the pandemic (*p* = 0.003). (Fig. 4**, **Table 5).

**Table 5:** Comparison between hospital admissions before and during COVID-19 pandemic

Hospital admissions	Before COVID-19 pandemic (*n* = 57)	During COVID-19 pandemic (*n* = 132)	*p*
Place			
Ward	24 (42.1%)	48 (36.4%)	0.456
ICU	33 (57.9%)	84 (63.6%)	
Cause			
COVID-19 infection	0 (0.0%)	60 (45.5%)	< 0.001*
Sepsis	9 (15.8%)	17 (12.9%)	0.594
CV events	10 (17.6%)	18 (13.6%)	0.594
Volume overload	15 (26.3%)	11 (8.3%)	0.001*
Vascular access complications	5 (8.8%)	11 (8.3%)	^FE^*p* = 1.000
Surgical/ interventions	8 (14.0%)	2 (1.5%)	^FE^*p* = 0.001*
Others	10 (17.5%)	13 (9.8%)	0.135
Duration (days)			
Median (IQR)	5.0 (3.0 – 5.0)	5.0 (4.0 – 7.0)	0.003*

*CV* cardiovascular, *IQR* interquartile range, *p* *p-*value for comparing between before COVID and after COVID group, *FE* Fisher Exact

*Statistically significant at *p* ≤ 0.05

### Predictors of dialysis adequacy

A multivariate regression analysis showed that the risk of inadequate dialysis defined as URR less than 65% increased with number of patients/nursing staff [OR: 1.508, CI: 1.274–1.786, *p* < 0.001] and mean IDWG [OR: 1.471, CI 1.277–102.348, *p* = 0.029] (Table 6).

**Table 6:** Multivariate logistic regression analysis for inadequate dialysis defined as URR < 65% regarding to different parameters after COVID (*n* = 324)

	OR (95%C.I)	*p*
Number of patients/nursing staff	1.508 (1.274–1.786)	< 0.001*
Mean IDWG	1.471 (1.104–1.961)	0.008*
Vascular access complications	1.583 (0.506–4.950)	0.430
No. of missed sessions	1.018 (0.974–1.064)	0.428
HB	1.008 (0.839–1.212)	0.928
Ca	1.099 (0.785–1.540)	0.581
PO4	1.104 (0.926–1.316)	0.272
PTH	1.000 (1.000–1.001)	0.420
Albumin	1.335 (0.739–2.412)	0.338

*OR* Odds ratio, *CI* Confidence interval, *LL* Lower limit, *UL* Upper Limit, *HB* hemoglobin, *Ca* calcium, *PO4* phosphorus, *PTH* parathyroid hormone, *p* *p*-value for Odds ratio for comparing between the studied groups

*Statistically significant at *p* ≤ 0.05

## Discussion

COVID-19 has had its toll on patients with chronic illnesses both directly and indirectly. Among our cohort of 388 HD patients from 5 HD units in Alexandria, Egypt, HD patient care was significantly affected during the first 10 months of the COVID-19 pandemic compared to the 10 months preceding the pandemic. Dialysis adequacy was reduced, other laboratory parameters showed evidence of poor nutrition as well as less control of anemia and CKD-MBD. There was also a higher rate of hospitalization and lower rate of elective procedures.

### Dialysis adequacy during the pandemic

Both URR and Kt/V as measures of dialysis adequacy were significantly reduced during the COVID-19 pandemic with lower levels during the initial lockdown and during the second wave of the pandemic. These measures were also lower during the months of curfew. In addition, there was a significant increase in the IDWG. These changes coincide with an increase in missed HD sessions, which reached its maximum level during the months of lockdown. Another reason may be the difficult accessibility to HD units reported by the 6% of patients during lockdown time.

Similarly, other studies reported reduced compliance with HD sessions during lockdowns. Prasad et al. [7] evaluated the impact of lockdown started by the government of India, to limit the spread of COVID-19 infection on the care of HD patients in 19 major hospital. The total number of patients coming for HD decreased from 2517 to 2404 and the number of serving dialysis machines declined from 523 to 496 after 3 weeks of lockdown. 28.2% of patients missed 1 or more HD sessions, 47% of these patients reported difficult accessibility to HD unit during the lockdown and 2.74% presented to the ER for emergency dialysis. A cross-sectional study including 80 HD patients from the Mounira children hospital in Cairo University reported that 12% of patients missed 1 or 2 HD sessions while 29% patients were delayed while going to the HD sessions due to transportation difficulties during the period from May 10th, 2020, to June 14th, 2020 [16]. Yet these studies did not examine the effect of poor accessibility and missed sessions on HD patient care.

Sousa et al. were the first team to examine the effect of the pandemic on HD patients, albeit in a single center including only 26 patients. They compared data at 2 points in time (1 month before and 1 month after the onset of the pandemic) and found a significant reduction in dialysis adequacy as assessed by URR% and Kt/V, both of which correlated with the duration of the dialysis sessions [17]. However, 1 month after the onset of the pandemic is too early to detect significant changes in the quality of care.

Contrarily, another study conducted on 30 HD patients in Indonesia, assessed Kt/V and IDWG during the pandemic and found them both adequate (2.04 and 2.25 kg, respectively) and concluded that the pandemic did not affect HD patient care, however, they did not compare the results to values before the pandemic [18]. The same group conducted another study assessing dialysis adequacy and IDWG in 105 dialysis patients over the course of 2 months during the COVID-19 pandemic. They did not observe significant change in dialysis adequacy or IDWG during that period, however, the values were again not compared to pre-pandemic levels. They also observed no deterioration of HRQoL as assessed by SF-36 questionnaire during these 2 months [19].

The multivariate regression analysis showed that the risk of having a URR of less than 65% increased with mean IDWG and reduced availability of nursing staff. Higher IDWG may reflect patients who missed sessions or who are non-compliant on dietary restrictions or patients without residual kidney function explaining its effect on adequacy. Our analysis supports the integral role of nursing staff on HD patients care. Similarly, a Korean nation-wide study explored the effect of nursing workload and years of experience on dialysis adequacy in 616 HD units. They found that an increase in the average daily number of HD cases per nurse led to significantly lower adequacy, but that the presence of nurses with ≥ 2 years of HD experience ameliorated this effect [20].

### Changes in dialysis care and access to HD units

Although patients in our cohort continued to be offered thrice weekly sessions, authors have debated the value of switching patients to twice weekly dialysis at this time of resource stress to preserve resources and staff and to limit exposure to potential infection in patients and staff. Twice weekly dialysis would allow better separation of patients and implementation of more vigorous infection control measures. Although this approach may be adequate in patients with residual renal function and dietary restrictions, it may not be appropriate for all HD patients [21–24]. This would have been a viable option had the restrictions imposed by the pandemic been a short-term emergency (rather than the repeated waves that followed). Others saw it as a possible last resort, that should not be done universally, but should rather be limited to patients with significant residual kidney function [24]. A possible alternative would be shortening the dialysis sessions. If twice weekly dialysis is contemplated, dietary restrictions should be advised to prevent hyperkalemia and volume overload between sessions with careful follow up [22].

Siga and a team in Argentina conducted a multi-center study to examine the efficacy of twice weekly HD during the pandemic. They initially chose patients with low ultrafiltration rate (< 8.5 ml/kg/h), patients without hyperkalemia and who also had a good nutritional status as assessed by Geriatric Nutritional Risk Index. These criteria were met by 50% of the patients in the studied units and was suggested as an option especially in COVID positive HD patients [25]. Based on their assessment of dialysis adequacy on this regimen, they added further restrictions to patients eligible to twice weekly dialysis excluding patients with urine output of less than 500 ml/day. They further noted that even in these patients, urea kinetic modelling should be closely monitored to assure adequate dialysis. Out of 110 patients in their study, only 22% achieved adequate dialysis with the twice weekly approach [23].

Another dilemma posed by the pandemic, and potentially with other disasters, is the increased need for acute dialysis. In-hospital HD units need to balance acute and prevalent dialysis patients. Carson et al. proposed an algorithm to allocate dialysis resources in case of future pandemics that relies on triaging patients based on IDWG and potassium and would allow prioritizing patients according to the urgency of their need for dialysis [26].

Another option is home dialysis (whether in the form of hemodialysis or peritoneal dialysis). Although it reduces the risk of spread of any communicable disease, it is a rather expensive option. Patients in remote areas will need continued monitoring and it is not suitable for frail elderly patients or patients living alone [27]. All these options and other potential plans should be contemplated and adapted to deal with any future pandemics or other disasters to ensure continued care for the vulnerable patients on hemodialysis.

### Clinical complications and comorbidities

Dialysis adequacy is just one aspect of HD patient care. Other aspects include healthy nutrition as well as anemia and CKD-MBD control. During the first 10 months of the pandemic, patients in our cohort had significantly lower hemoglobin level (*p* < 0.001), calcium level (*p* = 0.005) and albumin level (*p* < 0.001) and significantly higher phosphorus level (*p* = 0.033). This is consistent with results observed by Sousa et al. [17] who compared lab parameters 1 month before and 1 month after the pandemic. They found a significant reduction in red cell distribution width (*p* = 0.03), total proteins (*p* = 0.01); and albumin (*p* = 0.01), while phosphorus was significantly increased (*p* = 0.01). Some of these changes may be the result of poor adherence to medications, as erythropoiesis-stimulating agents, phosphate binders and vitamin D analogues. No studies specifically examined HD patient adherence to medications during the pandemic, but several studies reported poor adherence in other chronic diseases, partly because of closure of follow-up clinics, for fear of contracting COVID-19 during clinic or pharmacy visit or due to medication shortages [28, 29]. The utilization of telemedicine and the mobilization of community pharmacists to follow up patients with chronic diseases at time of physician shortages have been proposed to overcome some of these hurdles [29, 30].

The reduction in albumin and the elevation in serum phosphorus levels may be a consequence of a reduced quality of diet. The lockdown reduced the availability of fresh produce and led to reliance on processed foods which contain higher phosphorus. In fact, a systematic review of several longitudinal studies examining eating behavior during the COVID-19 pandemic reviewed 23 studies mostly conducted in general population cohorts. Pooled data revealed modified eating pattern with reduced adherence to healthy eating habits and increased consumption of sweets and ultra-processed foods [31].

### Impact on morbidity and hospitalization

Regarding hospitalization, there was a significant decrease in rate of surgical and elective interventions during the pandemic (*p* = 0.001) as well as a surprising significant decrease in the rate of patients presenting with inadequate HD and volume of overload during the pandemic (*p* = 0.001), while COVID-19 infection represented the most common cause for hospitalization in the pandemic period. The lower number of patients presenting with volume overload may be due to more caution regarding fluid intake taken by patients in order to miss sessions. Prasad et al. [7] reported that the attendance in the outpatient clinics decreased by 92.3%, and the inpatient service dropped by 61%. This does not necessarily reflect a lower need for hospitalization. It could reflect the fear of visiting health care facilities to avoid the risk of infection during the pandemic and difficult transportation during lockdown. A study by Cassell et al. examined the U.S. national healthcare billing database to review hospital admissions across the country before and after pandemic. They observed that the majority of primary diagnoses declined during the early months of the pandemic, with some illnesses returning to the pre-pandemic rates by late 2020 and early 2021 [32].

### Future directions

The COVID pandemic demonstrated that the world is not ready to deal with a global disaster. In time of disasters, provision of care should continue for patients with chronic diseases including HD patients. This is in line with last year’s world kidney day theme “Kidney Health for All–Preparing for the unexpected, supporting the vulnerable” [11]. Continued provision of dialysis supplies, preparing emergency plans for triaging patients requiring dialysis (whether acute or chronic dialysis cases) and increasing the scope of home dialysis may be ways to avoid such a reduction in the quality of care. Better patient education may also help in continued adherence to medications, the dialysis schedule and healthy nutrition.

### Strengths and limitations

There are several strengths to our study. It is the first study to compare dialysis adequacy over time during the pandemic. It is also a multi-center study with a large number of participants. In addition to adequacy, other measures of patient care were examined. However, there still were some limitations in this study. First, the retrospective design depends on previously recorded data and increases the risk of recall bias. Second, missing clinical data of the patients who died before the pandemic limited the comparison between the causes of mortality before and during the COVID-19 pandemic.

## Conclusions

In addition to the direct effect of COVID-19 pandemic on morbidity and mortality, the pandemic affected the quality of delivered care in HD units. During the COVID-19 pandemic, patients missed more sessions, the dialysis adequacy deteriorated, IDWG increased, hemoglobin, albumin and calcium decreased, while phosphorus and PTH levels increased. Better preparation for any future disasters is necessary to ensure continued optimal care of chronic patients.
